# Fructosamine and diabetes as predictors of mortality among Hispanic and non-Hispanic white breast cancer survivors

**DOI:** 10.1038/s41523-018-0099-x

**Published:** 2019-01-07

**Authors:** Avonne E. Connor, Kala Visvanathan, Stephanie D. Boone, Nader Rifai, Kathy B. Baumgartner, Richard N. Baumgartner

**Affiliations:** 1 Departments of Epidemiology and Oncology, Johns Hopkins Bloomberg School of Public Health and the Sidney Kimmel Comprehensive Cancer Center, Baltimore, MD USA; 2 Department of Epidemiology and Population Health and the James Graham Brown Cancer Center, University of Louisville, Louisville, KY USA; 3 Laboratory Medicine, Children’s Hospital Boston, Boston, MA USA

## Abstract

Epidemiologic studies have found that elevated insulin levels and chronic hyperglycemia among breast cancer (BC) survivors are associated with poor prognosis; few of these studies have included Hispanic women in whom diabetes is highly prevalent. We examined the associations between circulating fructosamine-a biomarker of hyperglycemia and blood glucose control, self-reported diabetes, and risk of BC-specific and all-cause mortality among Hispanic and non-Hispanic white (NHW) women diagnosed with invasive BC. A total of 399 BC survivors (96 Hispanic, 303 NHW) contributed baseline data and plasma samples. Hazard ratios (HR) and 95% confidence intervals (CI) were calculated using multivariable Cox proportional hazards regression models. After a median follow-up time of 13 years, a total of 134 deaths occurred, of which 56 deaths were from BC. Diabetes was associated with BC-specific (HR, 2.89; 95% CI 1.27–6.60) and all-cause (HR, 2.10; 95% CI 1.24–3.55) mortality. Associations were stronger among women with clinically high fructosamine levels (>285 µmol/L) (BC-specific: HR, 4.25; 95% CI 1.67–10.80; all-cause: HR, 2.32; 95% CI 1.30–4.14) compared to women with normal levels (≤285 µmol/L). In mediation analysis, fructosamine explained 47% of the association between diabetes and all-cause mortality and 41% of BC-specific mortality; the largest attenuation was among Hispanics for all-cause mortality (56%). Our results demonstrate that poor glycemic control explains a large extent of the relationship between diabetes and mortality among women with invasive BC, particularly among Hispanic women. The associations we observed for BC mortality should be confirmed in larger studies of ethnically diverse BC patients.

## Epidemiology: Poor glycemic control increases risk of cancer-linked death

Diabetes and poor blood sugar control significantly increase a woman’s odds of dying from invasive breast cancer. A team led by Avonne Connor from Johns Hopkins University in Baltimore, Maryland, USA, tracked the fate of 399 survivors of breast cancer, 96 of whom were Hispanic, an underrepresented ethnicity in many biomedical studies. After a median follow-up of 13 years, the researchers found that women with diabetes had a nearly 3-fold higher risk of dying from their breast cancer and more than a two-fold greater risk for all-cause mortality. Poor glycemic control, as measured by elevated levels of a common blood biomarker called fructosamine, explained a large extent of the relationship between diabetes and cancer-linked mortality, especially among Hispanic women. The findings suggest that better glucose management could improve outcomes for breast cancer survivors with diabetes.

## Introduction

Breast cancer is the leading cause of death among US Hispanic women, only surpassing heart disease within the last decade.^1,2^ According to the American Cancer Society, approximately 19,800 Hispanic women were diagnosed with breast cancer in the US in 2015, and approximately 2,800 women died from this disease.^2^ Evidence from epidemiologic studies primarily conducted in non-Hispanic white (NHW) women suggests that women with Type 2 diabetes (hereafter referred to as diabetes) and breast cancer may have an increased risk of breast cancer-specific^3–5^ and all-cause^6–8^ mortality. Few diabetes-breast cancer survival studies have included Hispanic women,^4,9^ in whom diabetes is highly prevalent.^10^

In a recent study including Hispanic and NHW breast cancer survivors from New Mexico, Utah, Colorado, Arizona, and San Francisco, we observed a 1.85-fold increased risk of breast cancer-specific mortality among Hispanic breast cancer patients with self-reported diabetes compared to Hispanic breast cancer patients without diabetes.^9^ Type 2 diabetes may influence breast cancer progression and outcomes through various pathways including the effect of high levels of insulin and insulin-like growth factors, sex hormones, and inflammation.^11^ In cell cultures hyperinsulinemia and hyperglycemia sequelae have been shown to increase tumor cell proliferation.^12^

Numerous studies have found that elevated insulin levels, hyperinsulinemia, and chronic hyperglycemia (measured by hemoglobin A1c/HbA1c) are associated with poor prognosis in women diagnosed with breast cancer;^11,13–15^ however, only one study, an earlier analysis of the Health, Eating, Activity and Lifestyle (HEAL) Study participants, has included US Hispanic women.^16^ A study of breast cancer patients in Mexico concluded that elevated blood glucose levels were associated with poor survival in diabetic and nondiabetic patients compared to patients with normal glycemic levels.^17^ In another study, high fasting blood glucose levels ( > 94 mg/dL), compared to those with glucose 84.1–94.0 mg/dL, were also found to be associated with increased risk of breast cancer-specific mortality among hormone receptor positive breast cancer patients.^18^

For the present study, we measured plasma fructosamine, a clinical measure of an individual’s average blood glucose control for the past 2 to 3 weeks,^19^ for assessment of hyperglycemia among Hispanic and NHW women previously diagnosed with invasive breast cancer from the New Mexico HEAL study. Fructosamine is a glycoprotein that results from the covalent attachment between a sugar to total serum proteins, and results from more rapid turnover of glycated proteins.^20^ We evaluated the prognostic significance of fructosamine with breast cancer-specific and all-cause mortality. To our knowledge, the relationship between fructosamine and breast cancer mortality has not been examined. Based on prior data presented, we postulated that Hispanic and NHW women with breast cancer that have chronic hyperglycemia, may have increased risk of breast cancer-specific and all-cause mortality. We also examined to what extent poor glycemic control might explain the relationship between diabetes and mortality after breast cancer, particularly among Hispanics.

## Results

After a median follow-up of 13 years since baseline interview, a total of 134 deaths occurred, of which 56 deaths were attributed to breast cancer. While more Hispanic women had diabetes (11.5%) compared to NHW women (7.6%), significant differences by ethnicity for fructosamine levels were not observed (Hispanic mean = 236 µmol/L; NWH mean = 238 µmol/L). Hispanics, however, had larger body size measures (body mass index (BMI), waist-hip ratio, percent body fat) at baseline interview compared to NHW survivors. Hispanic women were also significantly younger at diagnosis (Table 1). Among women with diabetes, 38% had clinically high fructosamine levels (>285 µmol/L).

**Table 1:** Baseline characteristics of the New Mexico Health Eating Activity and Lifestyle (HEAL) Study

Characteristics	Hispanic *N* = 96		Non-Hispanic white *N* = 303		
	Mean	SD	Mean	SD	*p*-value
Age at diagnosis (years)	54.8	11.8	60.2	12.5	<0.01
BMI at baseline (kg/m^2^)	27.2	5.8	25.7	5.4	0.02
Waist-hip ratio	0.86	0.07	0.83	0.07	<0.01
Percent (%) body fat	39.6	7.1	36.6	8.4	<0.01
Mean survival (years)	11.3	5	11.8	3.9	
Fructosamine (µmol/L)	235.7	41.1	237.6	31.5	0.67
History of diabetes	No.	%	No.	%	
No	85	88.5	280	92.4	0.24
Yes	11	11.5	23	7.6	
Clinically relevant fructosamine					
Normal (≤285 µmol/L)	92	96	285	94.1	0.51
High (>285 µmol/L)	4	5	18	5.9	
Median fructosamine					
<233 µmol/L	55	57.3	149	49.2	0.17
≥233 µmol/L	41	42.7	154	50.8	
Fructosamine quartiles					
<219 µmol/L	25	26.0	74	24.4	0.32
219–231 µmol/L	27	28.1	72	23.8	
232–251 µmol/L	24	25.0	77	25.4	
≥252 µmol/L	20	20.8	80	26.4	
Tumor characteristics	No.	%	No.	%	
Breast cancer stage					
Local	71	74	236	77.9	0.59
Regional	23	26	66	21.8	
Unknown	0	0	1	0.33	
Tumor subtype					0.12
Luminal A	33	34.4	144	47.5	
Luminal B	26	27.1	80	26.4	
HER2 overexpressing	10	10.4	19	6.3	
Triple-negative	11	11.5	20	6.6	
Missing	16	16.7	40	13.2	
Tumor size (cm)					
<1 cm	15	15.6	83	27.4	0.05
≥1 cm	78	81.3	210	69.3	
Unknown	3	3.1	10	3.3	
Lymph nodes positive					
All nodes negative	57	24.0	196	64.7	0.36
1–3 nodes positive	23	24.0	48	15.8	
≥4 nodes positive	3	3.1	19	6.3	
Positive, no. unspecified	0	0	1	0.33	
No nodes examined	13	13.5	38	12.5	
Unknown if positive	0	0	1	0.33	
Treatment	No.	%	No.	%	
Any chemotherapy	36	37.5	80	26.4	0.05
Surgery and radiation	31	32.3	31	45.2	
Surgery only	29	30.2	29	28.4	
Vital status					
Alive	66	68.8	199	65.7	0.58
Deceased	30	31.3	104	34.3	

In other descriptive statistics, we observed that women with diabetes were significantly an older age at breast cancer diagnosis (chi-sq. *p* = 0.001), and also had higher percent body fat (chi-sq. *p* = 0.01), higher body mass index (chi-sq. *p* < 0.001), and increased waist-hip ratio (chi-sq. *p* < 0.001) compared to women without diabetes; however, no significant differences were observed between women with a history of diabetes and without diabetes for breast cancer stage or treatment. These results were also not significantly different by ethnicity (data not shown).

As a continuous measure, fructosamine was positively associated with breast cancer-specific mortality (HR, 1.01; *p* = 0.01) and with all-cause mortality (HR, 1.01; *p* = 0.001) (Table 2). Associations were strongest among women with clinically high fructosamine levels (breast cancer mortality: HR, 4.25; 95% CI 1.67–10.80; all-cause: HR, 2.32; 95% CI 1.30–4.14) compared to women with normal levels (≤285 µmol/L) (Table 2). In analyses based on the median level, statistically significant associations were not observed among women with ≥ median (233 µmol/L) fructosamine levels (breast cancer mortality: HR, 1.03; 95% CI 0.59–1.82; all-cause: HR, 1.27; 95% CI 0.88–1.84); however models examining the associations by fructosamine quartiles were statistically significant for quartile 4 (≥252 µmol/L) compared to quartile 1 (<219 µmol/L) for all-cause mortality (HR, 2.08; 95% CI 1.25–3.46) but not for breast cancer mortality (HR, 1.62; 95% CI 0.71–3.69). For ethnic-stratified results with all-cause mortality, fructosamine as a continuous measure was significantly associated with increased risk of all-cause mortality among Hispanics (*p* = 0.0001) compared to NHW women (*p* = 0.26). Hispanic women with fructosamine levels in quartile 4 were also more than four times more likely to die of any cause (HR, 4.44; 95% CI 1.26–15.70); while no significant associations were observed among NHW women in this same risk group. Statistical power was limited to detect associations by ethnicity for breast cancer-specific mortality (Table 2). Significant statistical interactions for associations by ethnicity were not observed.

**Table 2:** The association between plasma fructosamine and mortality outcomes, the New Mexico HEAL Study

Fructosamine measures	All women (*N* = 399)			Hispanic (*N* = 96)			Non-Hispanic white (*N* = 303)		
All-cause mortality	Deaths	HR	95% CI	Deaths	HR	95% CI	Deaths	HR	95% CI
Clinically relevant cut-point									
Normal ≤ 285 µmol/L	115	1.00	Reference	26	1.00	Reference	89	1.00	Reference
High > 285 µmol/L	15	2.32	1.30–4.14	4	11.85	2.79–50.26	11	1.70	0.86–3.34
Continuous measure		1.01	*p* = 0.001		1.01	*p* = 0.001		1.00	*p* = 0.26
Median fructosamine									
<233 µmol/L	57	1.00	Reference	11	1.00	Reference	46	1.00	Reference
≥233 µmol/L	73	1.27	0.88–1.84	19	1.99	0.83–4.75	54	0.99	0.65–1.50
Quartiles									
<219 µmol/L	26	1.00	Reference	4	1.00	Reference	22	1.00	Reference
219–231 µmol/L	31	1.49	0.86–2.56	7	2.16	0.55–8.45	24	1.41	0.76–2.60
232–251 µmol/L	26	0.99	0.56–1.75	6	1.33	0.32–5.46	20	0.84	0.45–1.60
≥252 µmol/L	47	2.08	1.25–3.46	13	4.44	1.26–15.70	34	1.51	0.84–2.70
Wald test for trend			*p* = 0.03			*p* = 0.16			*p* = 0.28
Breast cancer-specific mortality									
Clinically relevant cut-point									
Normal ≤ 285 µmol/L	46	1.00	Reference	14	1.00	Reference	32	1.00	Reference
High > 285 µmol/L	6	4.25	1.67–10.80	2	15.17	2.03–113.38	4	2.71	0.84–8.70
Continuous measure		1.01	*p* = 0.01		1.01	*p* = 0.07		1.00	*p* = 0.42
Median fructosamine									
<233 µmol/L	29	1.00	Reference	8	1.00	Reference	21	1.00	Reference
≥233 µmol/L	23	1.03	0.59–1.82	8	0.78	0.26–2.37	15	0.84	0.42–1.68
Quartiles									
<219 µmol/L	11	1.00	Reference	3	1.00	Reference	8	1.00	Reference
219–231 µmol/L	18	1.85	0.85–4.01	5	2.53	0.51–12.52	13	1.95	0.77–4.93
232–251 µmol/L	10	1.03	0.42–2.51	4	1.06	0.79–6.02	6	0.90	0.30–2.71
≥252 µmol/L	13	1.62	0.71–3.69	4	1.11	0.22–5.61	9	1.38	0.51–3.75
Wald test for trend			*p* = 0.58			*p* = 0.38			*p* = 0.74

Models adjusted for age, BMI, stage, education, treatment, and ethnicity (among all women)

Self-reported diabetes history was also associated with increased breast cancer-specific mortality (HR, 2.89; 95% CI 1.27–6.60) and all-cause mortality (HR, 2.10; 95% CI 1.24–3.55) (Table 3), while results for breast cancer mortality should be interpreted with caution as only eight women with a history of diabetes died from breast cancer. Associations with all-cause mortality were stronger among Hispanics (HR, 3.07; 95% CI 1.05–8.94) compared to NHW women (HR, 1.67; 95% CI 0.86–3.24). In mediation analysis, adjusting for continuous fructosamine attenuated the associations with mortality outcomes among all women by 47% for all-cause mortality and 41% for breast cancer-specific mortality. The largest attenuation was observed among Hispanic women, as 56% of the association between diabetes and all-cause mortality was explained by hyperglycemia (Table 3).

**Table 3:** Associations between self-reported diabetes history and mortality and the role of fructosamine as a mediator, the New Mexico HEAL Study

Diabetes history	All women (*N* = 399)			Hispanic (*N* = 96)			Non-Hispanic white (*N* = 303)		
All-cause mortality	Deaths	HR	95% CI	Deaths	HR	95% CI	Deaths	HR	95% CI
None	109	1.00	Reference	22	1.00	Reference	87	1.00	Reference
Yes	21	2.10	1.24–3.55	8	3.07	1.05–8.94	13	1.67	0.86–3.24
% Explained by fructosamine^a^		1.57 (47%)	0.86–2.87		1.90 (56%)	0.61–5.96		1.52^b^	0.70–3.28
Breast cancer mortality	Deaths	HR	95% CI	Deaths	HR	95% CI	Deaths	HR	95% CI
None	44	1.00	Reference	13	1.00	Reference	31	1.00	Reference
Yes	8	2.89	1.27–6.60	3	2.52	0.59–10.79	5	2.18	0.73–6.52
% Explained by fructosamine^a^		2.09 (41%)	0.80–5.46		2.06^b^	0.58–7.34		1.73^b^	0.34–8.74

Models adjusted for age, BMI, stage, education, treatment, and ethnicity (among all women)

^a^Models adjusted for age, BMI, stage, education, treatment, ethnicity (among all women), and fructosamine (continuous)

^b^Percent (%) explained by fructosamine was not calculated since the associations for the main effects for diabetes and mortality outcomes were not statistically significant by ethnicity for breast cancer mortality and among NHW women for all-cause mortality

## Discussion

To our knowledge, the relationship between fructosamine and breast cancer mortality has not been previously examined. We measured plasma fructosamine for assessment of hyperglycemia among Hispanic and NHW women diagnosed with invasive breast cancer to evaluate the prognostic significance of this biomarker, in addition to self-reported history of diabetes, with all-cause and breast cancer-specific mortality. We observed that women with increasing fructosamine levels were at increased risk of both all-cause and breast cancer mortality. Women with clinically high levels were over two times more likely to die of any cause and over four times more likely to die of breast cancer, compared to women with normal fructosamine levels. Self-reported diabetes also significantly increased risk of mortality among breast cancer patients from our study; while the highest risk of death was observed among Hispanic women. Fructosamine was a strong mediator of the associations between diabetes and all-cause mortality, especially among Hispanics as nearly 60% of this association was attributed to blood glucose control.

One study did examine the association between fructosamine, serum insulin-like growth factor, platelet-derived growth factor, C-reactive protein and risk of breast cancer recurrence among 110 postmenopausal BC patients^14^; however, fructosamine was not associated with recurrence in this study population. Higher fructosamine levels have been reported to be associated with risk of overall mortality in people with cardiovascular disease.^21–24^

There is evidence that fructosamine is a good indicator of hyperglycemia in subjects with and without diabetes.^25^ Fructosamine is not affected by erythrocyte or hemoglobin characteristics, such as reduced blood cell age, lengthy blood cell survival, and disorders associated with blood cell survival,^20^ unlike HbA1c which is dependent on erythrocytes turnover and can only be measured in whole blood.^19^ Additionally, fructosamine is not affected by other factors that could modify HbA1c levels, such as cigarette smoking, alcohol and dietary fat consumption, liver diseases, age, iron deficiencies, and ethnic origin.^26^ While most of the women reported not having diabetes (91%) in our study sample, only 9 of these women might have undiagnosed diabetes based on high fructosamine levels (>285 µmol/L). In the study by Malmstrom et al.^25^, across glucose levels, strong correlations were found for fructosamine with glucose and HbA1c respectively, *r* = 0.75 and *r* = 0.78. Furthermore, fructosamine levels were a good discriminator between diabetics and nondiabetics (area under curve (AUC) = 0.91–0.95).

Higher levels of fructosamine have also been shown to predict type 2 diabetes.^19,26–29^ In a study conducted by Selvin et al.^19^ fructosamine levels in most cases correctly classified individuals into normal, prediabetic, or diabetic status based on HbA1c clinical cut-points. HRs for incident diabetes were 4.96 (95% CI, 4.36–5.64) for fructosamine above the 95th percentile and 1.97 (95% CI, 1.80–2.16) for fructosamine in the 75th to the 95th percentile; associations were attenuated but persisted after adjustment for HbA1c.^19^ Therefore, fructosamine appears to be a marker of hyperglycemia independent of HbA1c. Unfortunately, we were unable to compare the performance of both fructosamine and HbA1c in relation to survival in this study as the HEAL study does not have stored samples of whole blood.

Our study had strengths and additional limitations. Diabetes history was based on self-report and could be subject to recall bias. While we utilized only one measurement of fructosamine as a measure of hyperglycemia, this measurement was taken at the time of other baseline measures (on average 5 months post-diagnosis) and baseline lifestyle questionnaires. It is possible that breast cancer treatment could affect a patient’s diabetes status or glucose levels. However, among women that received chemotherapy treatment (*n* = 116), the majority were not under active treatment at baseline interview (94%). Breast cancer deaths were ascertained through the New Mexico SEER tumor registry which utilizes the underlying cause of death codified by ICD on death certificates. Issues with comparability and accuracy have been noted using these methods and could lead to potential misclassification of the underlying cause of death which could bias cancer-specific survival.^30^ However, some studies among SEER cancer registries have shown improved estimates in breast cancer-specific mortality when compared to other methods of estimating cause-specific death information.^31,32^ Furthermore, sample sizes and the number of events were small for breast cancer mortality associations and for stratifying models by ethnicity to asses if associations were of similar magnitude as results for all-cause mortality. Therefore, these results and potential associations should be interpreted with caution. Larger studies of Hispanic breast cancer survivors with diabetes are needed to confirm the associations between hyperglycemia and breast cancer outcomes among this minority population of women.

The HEAL study also captured various covariates of interest for this analysis, including numerous measures of body size, lifestyle variables, tumor prognostic characteristics, and breast cancer treatment. However, information pertaining to type of diabetic treatments was not collected. While metformin, an oral diabetes medication used to control blood sugar levels, has been found to be associated with reduced breast cancer risk and mortality,^33^ results for the associations between metformin and risk of breast cancer mortality among breast cancer patients with concurrent diabetes have been mixed.^34–37^ Future studies should also consider effects of diabetes medication use in relation to glucose control, especially among minority populations who might be more likely to have poor glycemic control with their diabetes.^38^

Our findings suggest that diabetes and fructosamine are significantly associated with increased risk of mortality among Hispanic and NHW women with invasive breast cancer. These associations are present more than 10 years post breast cancer diagnosis. The associations we observed for breast cancer-specific mortality were of similar magnitude to all-cause mortality but should be confirmed in larger studies of ethnically diverse breast cancer patients. Our results also demonstrate that poor glycemic control explains a large extent of the relationship between diabetes and mortality among women with breast cancer, particularly among Hispanic women. Interventions to reduce risk of poor outcomes among ethnically diverse breast cancer survivors should also consider methods to improve glycemic control among women with diabetes.

## Methods

### Study population

The New Mexico HEAL Study is a prospective case–cohort study conducted between 1996 and 1999. Details of the methods for subject selection, participation rates, data collection, and quality control procedures for the original HEAL study have been previously described.^39–41^ The primary objective of the HEAL study was to determine whether modifiable lifestyle factors (i.e., weight, physical activity, and diet), tumor hormone receptors, and other factors influence prognosis and if these associations differ by ethnicity. A total of 999 eligible first primary breast cancer cases were ascertained through the National Cancer Institute’s Surveillance Epidemiology and End Results (SEER) registry in New Mexico. Participation rates were higher among NHWs (65%) compared to Hispanics (55%).^39^ A total of 615 study participants, 18 years of age or older, diagnosed with first primary breast cancer (in situ to stage IIIA) between July 1996 and March 1999 were included in the parent study. Participants were residents of Bernalillo, Santa Fe, Sandoval, Valencia, or Taos counties at the time of diagnosis. Tumor characteristics and treatment information were abstracted from medical records. Baseline demographic characteristics, medical history, and lifestyle factors prior to diagnosis were collected approximately 5 months post-diagnosis by trained interviewers. A blood sample and anthropometric measurements were also collected at baseline interview. Ethnicity was based on self-report and was assessed at the time of screening for eligibility and at baseline interview. Written informed consent was obtained from each subject. The study was approved by the Human Research Protections Office at the University of New Mexico, in addition to the Institutional Review Board for Human Subjects at the University of Louisville.

**Fig. 1:**
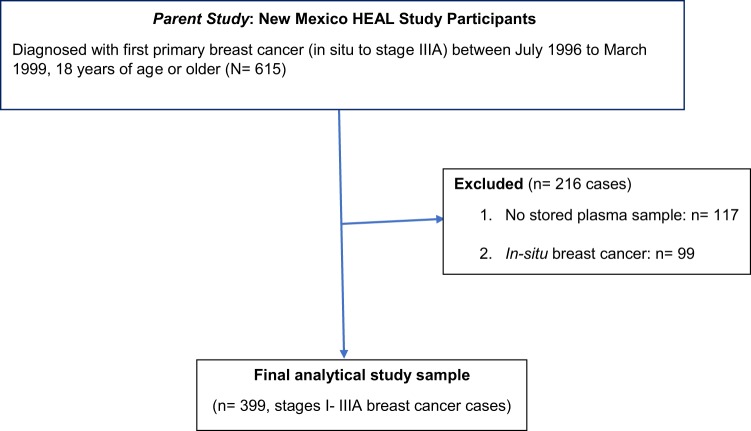
Inclusion criteria for the New Mexico Heath Eating Activity Lifestyle (HEAL) Study, fructosamine/diabetes survival analysis

Subjects who were diagnosed with invasive breast cancer (stages I–IIIA) and with a stored baseline plasma sample were eligible for inclusion in this analysis (*n* = 99 in situ cases excluded). A total of 399 invasive breast cancer survivors (96 Hispanic, 303 NHW) were included in the present study (Fig. 1).

### Fructosamine

The fructosamine assay was performed on the Roche P Modular system (Roche Diagnostics-Indianapolis, IN), using reagents and calibrators from Roche at the Rifai Laboratory (Boston Children’s Hospital). Lab investigators were blinded to any information about the samples Fructosamine was measured in 15 µL from approximately 600 aliquots of plasma samples collected at baseline interview, which have been in storage at −80 ^◦^C. Five percent of duplicates were assayed for quality control. The coefficient of variation (CV) was 1.31%. No samples were below the limit of detection for the HEAL study. Fructosamine levels were evaluated continuously, by median cut-point (median = 233 µmol/L), quartiles, and by clinically relevant cut-point (>285 µmol/L).^42^

### Assessment of diabetes history and covariates

Self-reported diabetes history (yes vs. no) and other covariates were obtained at the baseline interview. The following covariates were considered as potential confounders: age at diagnosis (continuous), body mass index (BMI, kg/m^2^) at interview (continuous) calculated by measured weight in kg and height meters, waist-hip ratio (WHR) (continuous), percentage of body fat measured by dual energy X-ray absorptiometry (DEXA) scans (continuous), education (high school, some college, college graduate/graduate school), tumor characteristics (i.e., stage of disease (local vs. regional); positive lymph node status (all nodes negative, 1–3 nodes positive, ≥4 node positive, positive but number unspecified, no nodes examined, unknown if positive); tumor subtype (luminal A, luminal B, HER2 overexpressing, triple-negative); tumor size (<1 cm vs. ≥1 cm) and treatment (any chemotherapy, surgery and radiation, surgery only).

### Assessment of study outcomes

Through linkage with the SEER cancer registry in New Mexico, we obtained information on vital status as of December 2013, including date of death or last follow-up (month and year) and cause of death. Survival (in years) was calculated as the difference between baseline interview date and date of death or last follow-up. For breast cancer-specific mortality, the cause of death was classified as breast cancer using the *International Classification of Diseases, 10th* *revision* (ICD-10) code C50.^43^

### Statistical analysis

Descriptive statistics were calculated by ethnicity for fructosamine levels (continuous, median cut-point, quartiles, and clinically relevant cut-point^42^), age at diagnosis, diabetes history, breast cancer stage, education, BMI, WHR, body fat percentage, and treatment, and chi-square tests and *t*-tests were used to compare ethnic groups. Adjusted hazard ratios (HRs) and 95% confidence intervals (CIs) were calculated by Cox proportional hazards regression models for associations with all-cause and breast cancer-specific mortality and by ethnicity. The proportional hazards assumption was tested statistically using an interaction of main effects and covariates with the log of survival time. The proportional hazards assumption was not violated for either outcome. Interactions between fructosamine measures and ethnicity were assessed using the likelihood-ratio test comparing the model including an interaction term with a reduced model without the term. A Wald test for linear trend was also conducted by assigning the median value of each fructosamine quartile category and modeling this variable as a continuous variable. Final models were adjusted for age, BMI, stage, education, treatment, and ethnicity (among all women).

To assess fructosamine as a mediator of the relationships between diabetes and survival outcomes, we compared a model that included all covariates and diabetes with a model that included the same variables and the fructosamine as a continuous variable. The percentage change in the HRs were then computed by the following formula:$\left[ {\left( {{\mathrm{HR}}_{{\mathrm{without}}\,{\mathrm{fructosamine}}}{\,-\,}{\mathrm{HR}}_{{\mathrm{fructosamine}}}} \right){\mathrm{/}}\left( {{\mathrm{HR}}_{{\mathrm{without}}\,{\mathrm{fructosamine}}}{ \,-\,} {{\mathrm1}}{\mathrm{.0}}} \right)} \right] \,\times\, {\mathrm{100;}}$ where HR_fructosamine_ denotes the HR for the effect of diabetes on breast cancer-specific and all-cause mortality after adjustment of fructosamine.^44^

A two-tailed probability value of <0.05 was considered significant for main effects and for interactions. All data analyses were performed using SAS version 9.4 (SAS Institute, Cary NC).

## Data Availability

The data that support the findings of this study are available on request from the senior author, (R.N. B.). The data are not publicly available due to restrictions with them containing information that could compromise research participant privacy.
